# Neglected yet pervasive: echinococcosis awareness and prevention capacity in Kyrgyzstan

**DOI:** 10.1017/S0031182024001343

**Published:** 2024-11

**Authors:** Dmitry Vishniakov, Mairamkul Turdumambetova, Nazgul Matkerimova, Kenesh Dzhusupov, Zildiz Sultanbaeva, Eldar Rafibekov

**Affiliations:** 1 Department of Public Health, International Higher School of Medicine, Bishkek, Kyrgyzstan; 2 Issyk-Kul Center for Disease Prevention and State Sanitary and Epidemiological Surveillance, Cholpon-Ata, Kyrgyzstan; 3 Department of Surgery, I.K. Akhunbaev Kyrgyz State Medical Academy, Bishkek, Kyrgyzstan

**Keywords:** Central Asia, echinococcosis awareness, echinococcosis prevention, echinococcosis, epidemiology of echinococcosis, Kyrgyzstan, practices related to echinococcosis, rural community

## Abstract

The study aimed to assess the heterogeneity in the distribution of disease awareness, attitudes, and practices related to cystic echinococcosis (CE) in different subgroups and inform health authorities regionally and globally for future evidence-based tailored prevention practices in the region. A cross-sectional study was conducted with 242 participants from Kyrgyz Republic (KR), Issyk-Kul oblast, and utilized survey data to analyse demographics, household information, echinococcosis-related practices, and knowledge. Participants in high-risk environments (HRE) and engaging in high-risk behaviours (HRB) linked to CE contracting were identified. Out of 242 participants, 39% lived in HRE, with 22% engaging in HRB of contracting CE. 13% lived in HRE and engaged in HRB. Only 6% followed all preventive measures, while 56% followed some. 97.5% of participants had heard about CE, but only 6% identified all transmission routes, and 63.4% were unaware of dog contact as a route. Education reduced the odds of being in the highest risk group (HRE&HRB) (OR 0.5, 95% CI 0.23–0.80). The study's findings are alarming, emphasizing factors contributing to regional endemicity. We anticipated a similar pattern in the neighbouring countries, given the shared nomadic customs and historical parallels. Examination of the heterogeneity of disease awareness and practices allows tailored prevention strategies. Urgent prevention programmes focusing on echinococcosis awareness in the KR are crucial to addressing challenges posed by nomadic habits.

## Graphical abstract

**Figure:**
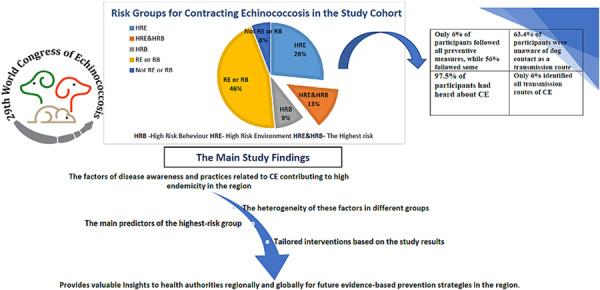

## Introduction

Echinococcosis is one of the 17 neglected tropical diseases (NTDs) stated by the World Health (World Health Organization, 2021). The disease affects more than 1 million people around the world and causes 19 300 deaths and around 871 000 disability-adjusted life-years globally each year (Agudelo Higuita, Brunetti and McCloskey, 2016; World Health Organization, 2021). The global distribution of echinococcosis has remained stable over the last 20 years, with consistent patterns of high endemicity in regions such as western China, Central Asia, South America, Mediterranean countries, and Eastern Africa (Craig *et al*., 2007; Wen *et al*., 2019). Thus, in areas with high endemicity, the annual incidence of cystic echinococcosis (CE) varies from less than 1 to 200 cases per 100 000 people (Wen *et al*., 2019; World Health Organization, 2021). Central Asia, and more specifically, the Kyrgyz Republic (KR), stands out as an endemic region with predictions of a significant increase in echinococcosis made a decade ago (Torgerson, 2013).

According to official statistics, echinococcosis remains a persistent and significant public health challenge in Kyrgyzstan (KR) (Department of Disease Prevention and State Sanitary and Epidemiological Surveillance under the Ministry of Health of the Kyrgyz Republic, no date; National Statistical Committee of the Kyrgyz Republic, no date). Over the past two decades, the incidence of echinococcosis has increased by 1.9 times, based on data from 2003 to 2022 (National Statistical Committee of the Kyrgyz Republic, no date). The average incidence rate over this period was 14.6 cases per 100 000 population, with the lowest incidence recorded in 2004 (9.2) and the highest in 2014 (20.2) (Raimkulov, 2020). Effective management in endemic regions focuses on control, prevention, and raising awareness about the disease and understanding relevant practices and attitudes within the affected population. Potential risk factors for contracting cystic echinococcosis (CE) are extensively examined in the literature and include dog-related, food-related, occupational, and socio-cultural factors (Wen *et al*., 2019; Altintas *et al*., 2021). While these risk factors are well-documented, their distribution varies due to regional biotic and abiotic differences (Possenti *et al*., 2016). Studying these risk factors is further challenged by the long incubation period of human CE and variations in regional behaviour and socioeconomic conditions (Possenti *et al*., 2016; Altintas *et al*., 2021). Previous studies examining risk factors for echinococcosis often treated the study population as a homogeneous group (Khan *et al*., 2021; Jamill *et al*., 2022; Lounis *et al*., 2023). Typically, these studies identified the highest risk group by occupation, such as butchers, or by specific environmental or behavioural factors, like having livestock at home or engaging in home slaughtering. Our study, however, adopted a different strategy by categorizing participants into three, not mutually exclusive groups: the high-risk environment group (HRE), which includes individuals living in high-risk environments of contracting CE; the high-risk behaviour group (HRB), consisting of individuals engaging in high-risk behaviours of contracting CE; and the highest risk group (HRE&HRB), which includes individuals both living in high-risk environments and engaging in high-risk behaviours. This approach enables a comprehensive understanding of population heterogeneity in high-prevalence CE areas, facilitating tailored prevention strategies for each group.

Echinococcosis presents a significant public health challenge in Kyrgyzstan, particularly in light of the WHO's goal to achieve disease control or elimination by 2050. Despite the dramatic changes and worsening situation with the disease in Central Asia, research on examining and monitoring CE risk factors has not received significant attention; there have been no publications in Central Asia in the past decade. Our study aimed to assess the heterogeneity in the distribution of disease awareness, attitudes, and practices related to CE in different subgroups. This study addresses a critical literature gap and provides valuable insights for health authorities regionally and globally for future evidence-based prevention policies and intervention strategies in the region.

## Materials and methods

### Study sample

We conducted a cross-sectional study during the ‘Month of Echinococcosis Awareness’ event in Issyk-Kul Oblast, Issyk-Kul region, KR, in April 2023. This region had the third highest prevalence rate of echinococcosis in KR with 13 cases per 100 000 of the population in 2020 (National Statistical Committee of the Kyrgyz Republic, no date). The survey was administered to rural authorities (Ail Okmotu) in 15 villages, teachers from a rural school in Grigorievka, residents of these villages, and the city, Cholpon-Ata. Out of the 247 distributed questionnaires, 242 participants returned completed forms, resulting in an impressive response rate of 98%. We deemed 5 questionnaires as unreadable and excluded them from the final sample.

Participation in the survey was voluntary, and the purpose of the study was thoroughly explained to all participants. They had the option to fill out either a paper-based or electronic version of the survey. No incentives were provided for participating, and personal identification information was not collected.

### Questionnaire content and validation

The self-administered questionnaire covered demographic characteristics, environmental factors, daily practices related to the possibility of CE contamination, and knowledge about CE. To ensure the questionnaire's reliability, it underwent validation in small groups and was tested in a pilot study.

### Identification of high-risk environments and high-risk behaviour

We employed a composite outcome variable with 2 levels (Yes/No) to identify HRE and HRB. HRE was assessed using questions related to participants' surroundings associated with CE risk. A score of 1 point was assigned if interviewees met specific criteria, such as having household members working with sheep or dogs, having close relatives or friends diagnosed with echinococcosis, living in a house with livestock, owning a dog, or witnessing stray dogs or cats around the house. Participants who obtained 4 or more points were considered to be living in an HRE. Table 2 contains the full list of questions and the distribution of study participants by the questions.

To assess high-risk behaviours (HRB) associated with the risk of contracting Echinococcosis (CE), we employed a set of 13 carefully crafted questions. These questions were designed to investigate participants' practices that may increase their susceptibility to CE infection.

The questions were divided into 2 categories: those connected to practices concerning dogs and those related to personal and household hygiene. For each question, participants who provided an affirmative answer were assigned 1 point, indicating engagement in a specific HRB.

Regarding practices connected to dogs, participants received 1 point for each of the following affirmative answers: never gave deworming tablets to their dog or neglected to take their dog to a veterinarian; never looked after neighbour's or stray dogs; had family members frequently petting or playing with the dog; owned a dog that consumed rodents; never kept their dog on a leash; fed their dog with raw meat and offal; often did not wash hands after contact with their dog; used dog feces as fertilizer or did not properly dispose of it. Similarly, questions linked to practices of personal and household hygiene included never washing hands with soap before eating or contacting their dog, consuming unwashed vegetables or drinking raw water, and engaging in home slaughtering and feeding dogs with cysts.

To compute the overall HRB score for each participant, we tallied the points obtained from these questions. If a participant accumulated 4 or more points, they were categorized as HRB. Table 3 contains the full list of questions and the distribution of study participants by the questions.

Finally, participants living in high-risk environments (HRE) and practicing high-risk behaviours (HRB) were identified as being in the highest-risk group (HRE&HRB).

Examining the study sample in these groups allows for a deeper understanding of the variations in the distribution of risk factors for echinococcosis. By recognizing the specific challenges and behaviours within groups, we can develop more targeted and effective prevention strategies tailored to the unique characteristics of each group.

### Statistical analysis

We employed descriptive statistics, *χ*^2^ tests, and logistic regression to explore socio-demographic factors influencing the group with the highest risk of contracting CE (participants in both groups HRE and HRB) and CE knowledge. Missing values were minimal, less than 3% across questionnaire sections, and did not pose any significant threat to our analysis. The highest percentage of missing data (less than 6%) was observed in the age variable, but we ensured there were no imbalances among the main outcome groups.

Logistic regression analyses independently examined the relationship between socio-demographic variables and the main outcome (HRB&HRE). A statistical significance level of 0.05 was applied, and the data analysis was conducted using SAS 9.04 software (SAS Institute).

## Results

The final analytical sample comprised 242 participants, with a slightly higher proportion of females (60.6%) compared to males (39.4%) (Table 1). The participants' mean age was 45.3 years (s.d. 13.0). The majority of respondents were married or in a common-law relationship (88.0%), with a smaller proportion being single or divorced (12.0%). In terms of their economic situation, 16.6% reported not being able to afford everything needed for a normal life, 60.2% indicated they could afford everything, and 23.2% reported being able to consume without any restrictions.

**Table 1.** Socio-demographic characteristics of the study participants

	Study sample Total *N* (col %)	*P* value	Living in a risky environment (HRE) *N* (col%)	Practicing risky behaviour (HRB) *N* (col%)	The highest risk (HRB and HRE) *N* (col%)
**Mean age of participants** (s.d.)	45.33 (13.02)		51.0 (7.07)	43.42 (13.11)	45.04 (13.66)
*Missing values N*	14				
Sex					
Male	95 (39.42)		40 (42.55)	19 (35.85)	11 (35.480)
Female	146 (60.58)	0.01	54 (57.45)	34 (64.15)	20 (64.52)
*Missing values N*	1				
Place of living					
City	43 (17.84)		3 (3.19)	9 (16.98)	0
Village	199 (82.16)	<0.001	91 (96.81)	44 (83.02)	31 (100)
*Missing values N*	0				
Marital status					
Single	18 (7.47)		7 (7.45)	4 (7.55)	2 (6.45)
Married, no children	16 (6.64)		8 (8.51)	5 (9.43)	3 (9.68)
Married, has children	192 (79.67)	<0.001	74 (78.72)	40 (75.47)	24 (77.42)
Civil marriage	4 (1.66)		2 (2.13)	1 (1.89)	0
Divorced	11 (4.56)		3 (1.24)	3 (5.66)	2 (6.45)
*Missing values N*	1				
Financial status					
Respondents were living in precarious conditions					
Respondents cannot afford everything needed for a normal life	40 (16.60)		17 (18.09)	12 (22.64)	7 (22.58)
Respondents can afford everything needed for a normal life	145 (60.17)	<0.001	61 (64.89)	33 (62.26)	19 (61.29)
Respondents can consume without any restrictions	56 (23.24)		16 (17.02)	8 (15.09)	5 (16.13)
*Missing values N*	1				
Education					
Secondary or less than secondary education	40 (16.60)		11 (11.7)	10 (18.87)	6 (19.35)
More than secondary education	90 (37.34)	<0.001	44 (46.81)	23 (43.39)	13 (41.94)
Higher education	111 (45.64)		39 (41.49)	20 (37.73)	12 (38.71)
*Missing values N*	1				
Occupation					
Working in office	68 (28.10)		23 (24.47)	9 (16.98)	4 (12.90)
Farmer	33 (13.64)		25 (26.60)	16 (30.19)	11 (35.48)
Seasonal worker	32 (13.28)	<0.001	15 (15.96)	6 (11.32)	6 (19.35)
Housewife	46 (19.01)		10 (10.64)	10 (18.87)	6 (19.35)
Working in school	45 (18.06)		18 (19.15)	9 (16.98)	4 (12.09)
Health worker	17 (7.05)		3 (3.19)	3 (5.66)	0
**Total**	242 (100)		94 (100)	53 (100)	31 (100)

Regarding educational attainment, 45.6% had higher education, while 37.3% had more than a secondary education, and 16.6% had secondary education or less. The majority of participants (28.1%) worked in an office, and a significant number were farmers and seasonal workers (26.9%). Additionally, the sample included 18.1% school teachers, 7.1% health workers and 19.0% housewives. The higher percentage of respondents with higher education or working in an office can be attributed to the survey's distribution among rural authorities (Ail Okmotu).

Out of the total participants, 84 (38.8%) were living in high-risk environments (HRE), while 53 (21.9%) were engaged in high-risk behaviours (HRB) (Table 1). The age difference was not statistically significant between the 2 groups. The distribution of sex and marital status did not differ significantly between the research sample and the HRB or HRE groups. As anticipated, the highest percentage of participants in the HRE group (96.8%) lived in rural areas. Comparing the total participants' distribution, the HRE group had a slightly higher proportion of respondents with low financial status (18.1% *vs* 16.1%) and a lower proportion with high financial status (17.0% *vs* 23.2%). Interestingly, the largest subgroup in both HRE and HRB were participants with more than secondary education (46.8 and 43.4%, respectively), despite being the fourth largest subgroup in the overall sample.

Examining the factors that determined risky living environments, we discovered intriguing insights. Initially, only 20% of respondents had jobs related to sheep or dogs, but this percentage increased to more than 40% in HRE group (Table 2). Similarly, 30% of participants in the research sample were either sick or knew someone with echinococcosis, however, this percentage surged to almost 40% in both the high-risk behaviours (HRB) and HRE groups. Notably, over half of the sample participants had dogs at home, with this proportion soaring to more than 80% in both risk groups, and an astonishing 90% of respondents reported observing stray dogs around their homes. Furthermore, three-quarters of the participants owned livestock.

**Table 2.** Environmental factors or participants' surroundings associated with the risk of contracting echinococcosis (HRE)

	Living in a risky environment (HRE) *N* (col%)	Practicing risky behaviour (HRB) *N* (col%)	The highest risk (HRB and HRE) *N* (col%)	Study sample Total *N* (col %)
Is your job or the job of your household members connected to sheep or dogs?				
Yes	40 (42.55)	15 (28.30)	14 (45.16)	49 (20.25)
No	54 (57.45)	38 (71.70)	17 (54.84)	192 (79.34)
*Missing values N* (*%)*				
Were you, close relatives, or friends diagnosed with Echinococcosis?				
Yes	37 (39.36)	21 (39.62)	12 (38.71)	73 (29.88)
No	57 (60.64)	32 (60.38)	19 (61.29)	169 (70.12)
*Missing values N (%)*				
Are you living in apartments or your own house?				
Apartment	0	3 (5.66)	0	26 (10.74)
Own house	94 (100)	50 (94.34)	31 (100)	216 (89.26)
*Missing values N (%)*				
Do you have livestock?				
Yes	89 (94.68)	41 (77.36)	29 (93.55)	180 (74.69)
No	5 (5.32)	12 (22.64)	2 (6.45)	62 (25.31)
*Missing values N (%)*				
Do you have a dog at home?				
Yes	84 (89.36)	45 (84.91)	30 (96.77)	124 (51.24)
No	10 (10.64)	8 (15.09)	1 (3.23)	118 (48.76)
*Missing values N (%)*				
How often do you see strain dogs or cats around your house?				
All the time	27 (28.72)	21 (39.62)	11 (35.48)	56 (23.24)
Sometimes	63 (67.02)	30 (56.60)	20 (64.52)	163 (67.63)
Never	4 (4.26)	2 (3.77)	0	23 (9.13)
*Missing values N (%)*				
**Total**	94 (100)	53 (100)	31 (100)	242 (100)

The investigation of daily practices related to Echinococcosis contamination involved 2 sets of questions: those concerning dogs and those associated with personal and household hygiene (Table 3).

**Table 3.** Practices connected to the possibility of contamination or contracting echinococcosis

	Living in a risky environment (HRE) *N* (col%)	Practicing risky behaviour (HRB) *N* (col%)	The highest risk (HRB and HRE) *N* (col%)	Study sample Total *N* (col %)
Do you practice home slaughtering?				
Yes	21 (22.34)	18 (33.96)	14 (45.16)	31 (12.86)
No	73 (77.66)	35 (66.04)	17 (54.84)	210 (87.14)
*Missing values N (%)*				1
What did you do with cysts?				
Burn	25 (26.60)	3 (5.66)	3 (9.68)	71 (29.46)
Bury or throw away	68 (72.34)	49 (92.45)	27 (87.1)	167 (69.29)
Feed dogs	1 (1.06)	1 (1.89)	1 (3.23)	3 (1.24)
*Missing values N (%)*				1
Did you give your dog deworming tablets or show your dog to a veterinarian?				
All the time	31 (38.75)	12 (26.67)	9 (30.00)	37 (31.09)
Sometimes	35 (43.75)	18 (40.00)	11 (36.67)	61 (51.26)
Never	14 (17.50)	15 (33.33)	10 (33.33)	21 (17.65)
*Missing values N (%)*				5
Have you ever looked after your neighbour's or stray dogs?				
Yes	23 (24.47)	19 (35.85)	14 (45.16)	34 (14.11)
No	70 (74.47)	34 (64.15)	17 (54.84)	206 (85.890)
*Missing values N (%)*				2
Do you or your family members pet or play with your dog often?				
Yes	15 (15.96)	17 (32.08)	8 (25.81)	29 (12.03)
No	79 (84.04)	36 (67.92)	23 (74.19)	213 (87.97)
What do you feed your dog?				
Raw meat	10 (15.38)	8 (20.00)	5 (20.00)	15 (14.56)
Offal	7 (10.77)	7 (17.50)	4 (16.00)	12 (11.65)
Other	48 (73.85)	25 (62.50)	16 (64.00)	76 (73.79)
*Missing values N*				11
Does your dog eat rodents?				
Yes	19 (22.89)	13 (28.89)	9 (30.00)	**31** (**25.20)**
No	53 (63.86)	26 (57.78)	18 (60.00)	74 (60.16)
Do not know	11 (13.25)	6 (13.33)	3 (10.00)	18 (14.63)
*Missing values N*				1
What do you do with your dog's feces?				
Burn	18 (22.78)	17 (38.64)	9 (31.03)	36 (30.25)
Throw away	22 (27.85)	6 (13.64)	4 (13.79)	34 (28.57)
Use as fertilizer	4 (5.06)	2 (4.55)	1 (3.45)	5 (4.25)
Do nothing	35 (44.30)	19 (43.18)	15 (51.72)	44 (37.97)
*Missing values N*				5
Do you keep your dog on a chain?				
All the time	45 (55.56)	13 (29.55)	10 (34.48)	59 (48.76)
Sometimes	9 (11.11)	4 (9.09)	2 (6.90)	15 (12.40)
Never	27 (33.33)	27 (61.36)	17 (58.62)	47 (38.84)
*Missing values N*				3
Do you wash your hands with soap before eating?				
All the time	89 (94.68)	46 (86.79)	27 (87.10)	230 (95.44)
Sometimes	4 (4.26)	6 (11.32)	3 (9.68)	10 (4.15)
Never	1 (1.06)	1 (1.89)	1 (3.23)	1 (0.41)
*Missing values N*				1
Do you wash your hands after contact with a dog?				
All the time	92 (97.87)	48 (90.57)	29 (93.55)	233 (96.68)
Sometimes	1 (1.06)	4 (7.55)	1 (3.23)	8 (2.90)
Never	1 (1.06)	1 (1.89)	1 (3.23)	1 (0.410)
Do you eat unwashed vegetables?				
All the time	7 (7.45)	4 (7.55)	3 (9.68)	14 (5.81)
Sometimes	36 (38.30)	28 (52.83)	14 (45.16)	115 (47.30)
Never	51 (54.26)	21 (39.62)	14 (45.16)	113 (46.89)
Do you drink tap water?				
All the time	29 (30.85)	14 (26.42)	9 (29.03)	52 (21.58)
Sometimes	51 (54.26)	30 (56.60)	16 (51.61)	148 (61.00)
Never	14 (14.89)	9 (16.98)	6 (19.35)	42 (17.43)
**Total**	94 (100)	53 (100)	31 (100)	242 (100)

Of the 124 participants with dogs at home, a mere 31% regularly gave deworming medication to their pets, 26% fed dogs raw meat or offal, and this percentage rose to 36% in the HRB and HRE groups. Disturbingly, 42% did not appropriately dispose of their dogs' feces, often petting dogs (12%) or looking after stray dogs (14%) with even higher percentages observed in the HRE and HRB groups.

Conversely, when it comes to personal and household hygiene, the results were more promising, particularly concerning handwashing. Thus, 95% of respondents washed their hands with soap before meals or when in contact with their dogs (96.7%). However, approximately 53% of participants sometimes or regularly consumed unwashed vegetables, and 21.6% constantly drank raw water. Household practices linked to livestock also showed room for consideration as 12.9% engaged in home slaughtering, and only 30% properly disposed of CE cysts.

In terms of awareness, the majority of participants (97.9%) had heard about echinococcosis, with only a mere 5 respondents having no knowledge of the disease (Table 4). Nonetheless, it is concerning that only 40% identified dogs as a possible source of CE transmission, and merely 17% correctly identified all infection sources.

**Table 4.** Echinococcosis awareness in the study population

	Living in a risky environment (HRE) *N* (col%)	Practicing risky behaviour (HRB) *N* (col%)	The highest risk (HRB and HRE) *N* (col%)	Study sample Total *N* (col %)
Have you ever heard about echinococcosis?				
Yes	93 (98.94)	50 (94.34)	30 (96.77)	236 (97.93)
No	1 (1.06)	3 (5.66)	1 (3.23)	5 (2.07)
*Missing values N (%)*				0
If you have heard of echinococcosis, then how can you get infected with it?				
Contact with dog	28 (33.73)	16 (32.00)	7 (23.33)	88 (39.54)
Contact with the skin of animals	14 (16.87)	15 (30.00)	6 (20.00)	75 (33.78)
Eat liver, lungs with cysts domestic animals	58 (69.88)	37 (26.00)	24 (80.00)	176 (79.28)
Contact with sick people	36 (43.37)	25 (50.00)	16 (53.33)	98 (44.14)
Unwashed food or tap water	36 (43.37)	25 (50.00)	16 (53.33)	26 (11.71)
Do not know	2 (2.17)	2 (3.77)	1 (3.23)	5 (2.10)
*Missing values N (%)*				4

Examination of the socio-demographic characteristics of participants revealed 2 statistically significant covariates that might predict participants at the highest risk of contracting CE (participants in both groups HRE and HRB). Thus, respondents with higher education had a 46% lower chance of being in the risk group compared to those who had more than secondary education (OR 0.54 (95% CI 0.27–0.98). Participants who worked in an office had a 78% less chance of being in the highest risk group compared to farmers (OR 0.22 (95% CI 0.05, 0.91). However, the model with all socio-demographic predictors did not show statistically significant predictors.

**Figure 1.**
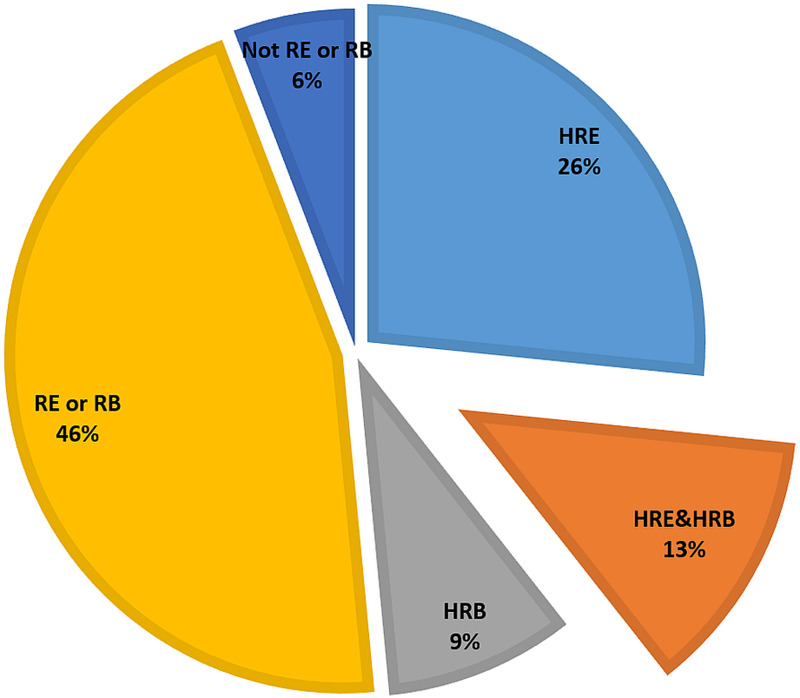
The distribution of study participants living in risky environments or practicing risky behaviours associated with contracting echinococcosis. RE, risky environment; RB, risky behaviour; HRE, high risky environment; HRB, high risky behaviour.

Figure 1 presents the overall landscape of the distribution of study participants living in risky environments or practicing risky behaviours associated with contracting CE in the region. Out of 242 participants, 39% lived in HRE and 22% engaged in HRB of contracting CE. 13% lived in HRE and engaged in HRB. 46% of the study population obtained 3 or fewer points on living in a risky environment (RE) or engaging in risky behaviour (RB) and only 6% followed all preventive measures.

## Discussion

The Kyrgyz Republic (KR) is a landlocked country predominantly inhabited by people of Kyrgyz nationality, historically characterized by nomadic traditions. Notably, the incidence of the disease has nearly doubled over the past decade (Department of Disease Prevention and State Sanitary and Epidemiological Surveillance under the Ministry of Health of the Kyrgyz Republic, no date; National Statistical Committee of the Kyrgyz Republic, no date). Surprisingly, our research did not find recent studies with the primary goal of assessing disease awareness and practices related to CE in the Central Asia region. Given the shared nomadic customs and historical parallels in the development of neighbouring countries, it's reasonable to anticipate similar patterns. The findings from our study shed light on the potential scope of disease awareness and practices concerning CE, as well as the factors contributing to its high endemicity in the region.

Despite all advances in the diagnosis and treatment of echinococcosis (Wen *et al*., 2019) the main key in disease management is prevention, and disease awareness plays a crucial role in it (Cvejic *et al*., 2016). The study sample's knowledge about echinococcosis could be described as a mixture of awareness and uncertainty. While the majority of participants (98.94%) had heard about echinococcosis, only a few truly understood what it entails. These results might look very promising as other researchers reported that less than 50% of the population living in highly endemic areas ever heard about zoonosis (Qucuo *et al*., 2020; Khan *et al*., 2021; Jamill *et al*., 2022). Yet, merely 40% correctly identified dogs as a potential source of CE transmission, and a mere 17% were able to identify all possible infection sources accurately. These findings underscore the importance of targeted awareness campaigns and education initiatives to bridge the gap between knowledge and understanding of echinococcosis.

When examining practices linked to the risk of contamination or contracting echinococcosis, there were both encouraging and concerning findings. Personal hygiene, particularly handwashing, showed promising results. Household practices associated with livestock also raised some concerns. A significant percentage admitted to engaging in home slaughtering, and only 30% of participants properly disposed of CE cysts, indicating the need for improvement in these areas. The results are consistent with other studies, showing a slightly higher percentage of adherence among the Kyrgyz population (Qucuo *et al*., 2020; Khan *et al*., 2021; Jamill *et al*., 2022; Lounis *et al*., 2023). Given the concerning findings, authorities must take strict measures against home slaughtering practices.

The most alarming findings came to light when examining practices related to dogs. Among the dog owners, a mere one-third of respondents regularly administered deworming medication to their dogs, and a quarter of participants fed their dogs raw meat or offal. Disturbingly, this percentage increased in the HRB and HRE groups, indicating the persistence of risky behaviours among those at higher risk. Moreover, dog owners did not appropriately dispose of their dog's feces, and a considerable number reported petting or looking after stray dogs, further increasing the risk of exposure to echinococcosis, particularly in the HRE and HRB groups. The literature review revealed a range of population commitments to these practices, with prevalence largely dependent on whether the study samples were predominantly rural or urban (Qucuo *et al*., 2020; Lounis *et al*., 2023). The study highlighted the importance of strengthening dog management. Encouraging regular deworming of dogs and proper disposal of their feces can significantly reduce the risk of infection. Local authorities and veterinarians should collaborate to provide accessible and affordable deworming services to dog owners.

While higher education initially reduces the odds of being in the highest-risk group by 50%, this effect was not statistically significant after adjusting for other socio-demographic characteristics in the model. Therefore, these characteristics cannot be used as a reliable predictor for identifying participants in the highest-risk group. Recent studies examining the impact of education on disease awareness and practices did not identify any association (Qucuo *et al*., 2020; Khan *et al*., 2021; Jamill *et al*., 2022; Lounis *et al*., 2023).

The majority of studies that examined awareness and practices related to CE often considered the study population as a homogeneous group distinguishing only between urban and rural residences (Khan *et al*., 2021; Jamill *et al*., 2022; Lounis *et al*., 2023). However, these broad categories encompass diverse subgroups with varying distributions of CE risk factors. Our three-group approach reveals these differences, providing a better understanding of the population at risk of CE. By adopting this method, we delved into the diverse characteristics of the study population. This strategy not only pinpointed the socio-demographic differences of these groups but also facilitated the customization of prevention policies and interventions to address specific group distinctions.

Furthermore, it's worth noting that the main research sample primarily comprises the most vulnerable rural population, accounting for 82.16% of the participants. However, it's essential to acknowledge that the questionnaire distribution was mainly focused on the rural ‘elite,’ including rural authorities, teachers, and health workers, who made up nearly 50% of the respondents from rural areas. Additionally, only 39% of the research sample resided in high-risk environment (HRE) areas. Due to this sampling approach, there is a possibility that the study's findings might underestimate the overall assessment of echinococcosis awareness and practices within the general rural population.

Other limitations of the study arose from the nature of the survey. Our cross-sectional study collected self-reported data that were not validated against any records and were prone to response and social desirability biases. Another limitation was 6.25% of participants that did not provide information about age. However, sensitivity analysis showed no imbalances of missing values among the main demographic and outcome groups. Other missing values did not exceed 3% of the total research sample and could not provide any threats to our study results.

## Conclusion

Our study explores disease awareness and practices related to CE, highlighting factors contributing to its regional endemicity. Given similar nomadic customs and historical development among neighbouring countries, we can expect comparable patterns in the region.

Our categorization of the study population into 3 groups (HRB, HRE and HRE&HRB) enables an exploration of its heterogeneity, leading to a deeper understanding of diverse characteristics and facilitating tailored prevention strategies based on specific group differences.

The study's primary findings are cause for concern regarding the future of echinococcosis in KR. Developing evidence-based policies and intervention strategies might be crucial to prevent the spread of the disease.

## Data Availability

The data that support the findings of this study are available from the corresponding author upon reasonable request.
